# Induction of human fetal hemoglobin expression by adenosine-2’,3’-dialdehyde

**DOI:** 10.1186/1479-5876-11-14

**Published:** 2013-01-14

**Authors:** Yinghong He, Gerhard Rank, Miaomiao Zhang, Junyi Ju, Ronghua Liu, Zhen Xu, Fiona Brown, Loretta Cerruti, Chi Ma, Renxiang Tan, Stephen M Jane, Quan Zhao

**Affiliations:** 1 Molecular Immunology and Cancer Research Center, The State Key Laboratory of Pharmaceutical Biotechnology, School of Life Sciences, Nanjing University, Nanjing, 210093, China; 2 Department of Medicine, Monash University Central Clinical School, Alfred Hospital, Prahran, VIC, 3181, Australia; 3 School of Basic Medicine, Dali University, Yunnan, 671000, China

**Keywords:** Adenosine-2’,3’-dialdehyde (Adox), Fetal globin, Histone arginine methylation, DNA methylation

## Abstract

**Background:**

Pharmacologic reactivation of fetal hemoglobin expression is a promising strategy for treatment of sickle cell disease and β-thalassemia. The objective of this study was to investigate the effect of the methyl transferase inhibitor adenosine-2’,3’-dialdehyde (Adox) on induction of human fetal hemoglobin (HbF) in K562 cells and human hematopoietic progenitor cells.

**Methods:**

Expression levels of human fetal hemoglobin were assessed by northern blot analysis and Real-time PCR. HbF and adult hemoglobin (HbA) content were analyzed using high-performance liquid chromatography (HPLC). DNA methylation levels on human gamma-globin gene promoters were determined using Bisulfite sequence analysis. Enrichment of histone marks on genes was assessed by chromosome immunoprecipitation (ChIP).

**Results:**

Adox induced γ-globin gene expression in both K562 cells and in human bone marrow erythroid progenitor cells through a mechanism potentially involving inhibition of protein arginine methyltransferase 5 (PRMT5).

**Conclusions:**

The ability of methyl transferase inhibitors such as Adox to efficiently reactivate fetal hemoglobin expression suggests that these agents may provide a means of reactivating fetal globin expression as a therapeutic option for treating sickle cell disease and β-thalassemia.

## Background

Human globin genes undergo two switches during development, from embryonic (ε) to fetal (γ) and from fetal (γ) to adult (β) globin [1]. Reactivation of fetal hemoglobin (HbF) in adults is one of the most effective strategies for treatment of sickle cell disease and β-thalassemia as increased fetal globin levels are associated with improved symptoms in hemoglobinopathy patients [2].

To date, compounds such as 5-aza-2-deoxycytidine (decitabine) [3], hydroxyurea (HU) [4], short-chain fatty acids (SCFAs) [5], and histone deacetylase (HDAC) inhibitors [6], have been used to increase HbF, although their effects are variable. However, the mechanism of action is not clear and remains controversial [2].

In previous studies, we determined that protein arginine methyltransferase 5 (PRMT5) coupled with Dnmt3a and related complexes played a critical role in human fetal globin gene repression [7,8]. In this report, we have found that treatment with adenosine-2’,3’-dialdehyde (Adox), a methyltransferase inhibitor [9,10], can induce fetal hemoglobin gene transcription in adult human bone marrow erythroid progenitor cells. This activity of Adox may be associated with inhibition of PRMT5.

## Methods

### Cell culture and reagents

K562 cells were cultured as described previously [7]. To generate human bone marrow (BM) erythroid progenitor cells, isolated CD34+ cells were grown in StemSpan SFEM medium with 1X CC100 cytokine mix for 6 days, then reseeded into the same medium supplemented with SCF (20 ng/ml), EPO (1 U/ml), IL-3 (5 ng/ml), dexamethasone (2 μM), and β-estradiol (1 μM), and cultured for two more weeks [11]. Cell surface marker analysis with CD71 and Glycophorin A indicated that the cultured cells were greater than 90% erythroid lineage. Human BM cells were collected under approval by the Melbourne Health Human Research Ethics Committee.

A 25 mM stock solution of adenosine-2’,3’-dialdehyde (Adox, Sigma) was prepared in 0.04 M HCl. Decitabine (Sigma) was prepared in phosphate-buffered saline (PBS). Working solutions of both reagents were prepared by dilution in PBS immediately prior to use.

### Benzidine staining, Northern Blot, and DNA methylation analysis

K562 cells were treated with Adox (2.5 μM) for 2 days and washed twice with ice-cold phosphate-buffered saline. The cell pellets were then resuspended in ice-cold phosphate-buffered saline. Benzidine solution (0.1% 3,3’-diaminobenzidine containing hydrogen peroxide) was added at room temperature. Benzidine-positive cells were spun onto a glass plate, examined by light microscopy and photographed. Northern blot analysis of K562 cells was performed as described previously [12]. Bisulfite sequence analysis was performed as described previously [7]. PCR was performed with HiFi Taq polymerase (Roche) as follows: 30 cycles, 94°C for 20 s, 55°C for 20 s, and 68°C for 35 s. PCR products were cloned into pCRII (Invitrogen) followed by nucleotide sequencing using the Big-Dye Termination method (ABI). At lease 40 clones were sequenced for each CpG site.

### ChIP analysis

ChIP assays were performed as described previously [7]. H4R3me2s and histone H4 acetylation antibodies were purchased from Abcam. Each experiment was performed independently at least twice. The ChIP samples were analyzed by quantitative real-time PCR using FastStart Universal SYBR Green Master (Roche). A standard curve was prepared for the primers using serial titration of the input DNA. The percentage of ChIP DNA was calculated relative to the input DNA from primer-specific standard curves using Rotor-Gene 6000 Series Software 1.7.

### Quantitative RT-PCR (Q-RT-PCR)

Total RNA was isolated from cells with Trizol reagent (Invitrogen). cDNA was generated using a reverse transcription system (Promega). The identities of the amplified bands were confirmed by sequencing. The PCR conditions and primers were described previously [7], and all samples were run in triplicate.

For bone marrow samples, plasmid DNA encoding γ-globin, β-globin or α-globin was used to generate the standard curve for determination of copy number. The number of molecules per nanogram total RNA from bone marrow cells was calculated from standard curves using Rotor-Gene 6000 Series Software 1.7.

### HPLC for adult and fetal hemoglobin

Aliquots of one million cells were washed in phosphate-buffered saline. The pellets were lysed by repeated freeze-thaw. The supernatant was analyzed for HbF and adult hemoglobin (HbA) content by ion-exchange high-performance liquid chromatography (HPLC) using a Bio-Rad VARIANT β-thalassemia Short Program.

## Results and discussion

**Figure 1:**
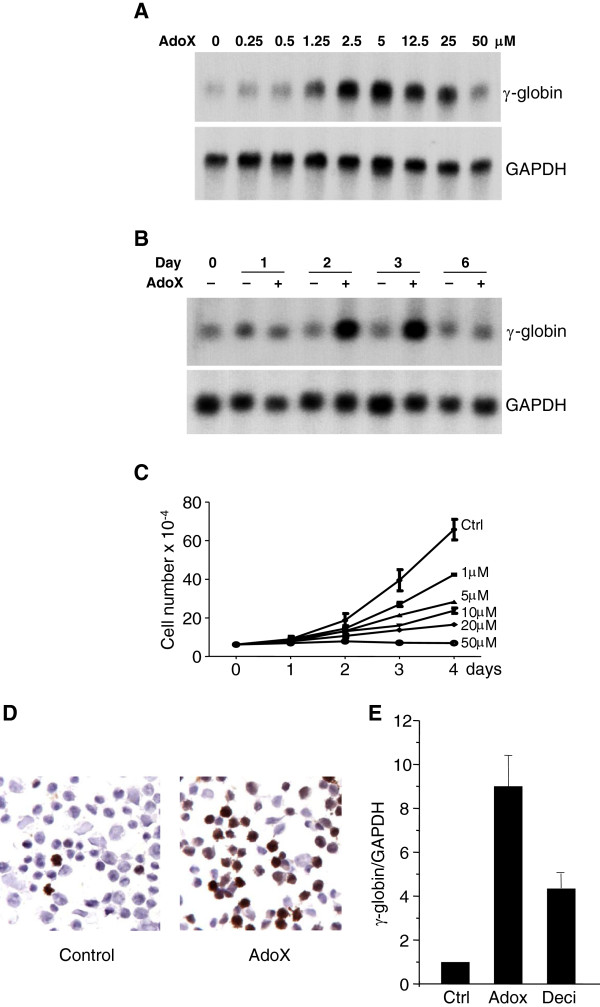
**Adox induced γ-globin gene expression in K562 cells.** (**A**) Northern blot analysis of total RNA from K562 cells treated with indicated concentrations of Adox for 2 days. (**B**) Northern blot analysis of total RNA from K562 cells treated with Adox (2.5 μM) for the indicated number of days. (**C**) Effects of Adox on in vitro growth of K562 cells. K562 cells were treated with the indicated concentrations of Adox or PBS control (Ctrl) and cell number/ml was determined at the indicated time. (**D**) Benzidine staining of K562 cells treated with Adox (2.5 μM) or PBS control (Ctrl) for 2 days. (E) Q-RT-PCR quantification of γ-globin from K562 cells treated with PBS control (Ctrl), Adox (2.5 μM) for 2 days, or decitabine (Deci, 4 μM) for one week (refreshed once after 3 days treatment). Graphs show mean ± SD, n = 3.

In order to test the effect of Adox to induce γ-globin, K562 cells were treated and showed a dose response effect on activation (Figure 1A). Next, we performed a time-course analysis of γ-globin induction by Adox. We found that from day 2 γ-globin expression was readily detected, but after day 6, induction stopped (Figure 1B). This result suggested that Adox could induce γ-globin very quick and it could also be metabolized during cell proliferation. Adox also induced a dose-dependent inhibition of in vitro proliferation of K562 cells,similar to the effect of decitabine (Figure 1C) [13]. Benzidine staining of K562 cells also showed activation effect of Adox on γ-globin (Figure 1D). Q-RT-PCR analysis further confirmed a 9-fold induction of γ-globin gene expression by Adox compared to the control (Figure 1E).

**Figure 2:**
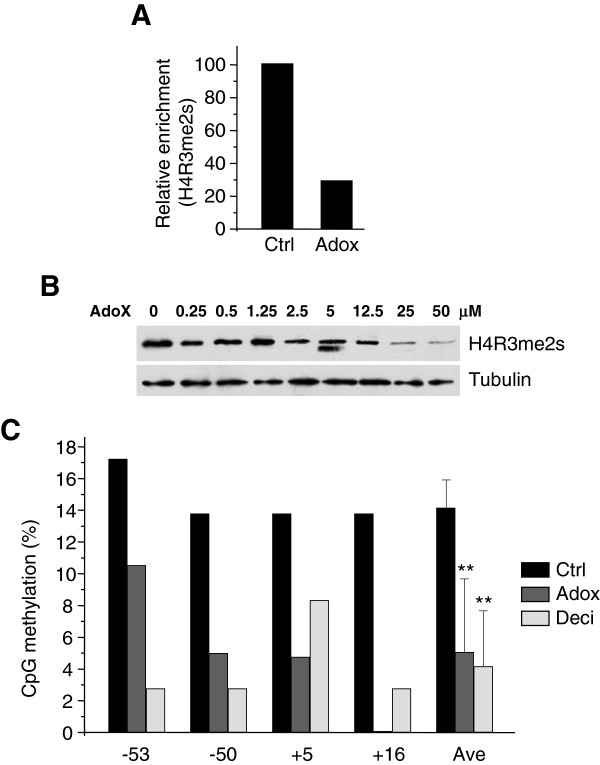
**Modulation of DNA methylation and histone methylation by Adox in K562 cells.** (**A**) Histone H4R3me2s ChIP analysis of γ-globin promoter from K562 cells treated with Adox or PBS control. Graphs show mean ± SD, n = 3. (**B**) Western blot analysis of proteins with histone H4R3me2s and anti-tubulin antibodies from K562 cells treated with indicated concentrations of Adox for 2 days. (**C**) Quantification of DNA methylation at the human γ-globin gene in K562 cells treated with Adox, decitabine (Deci), or PBS control as in Figure 1E. Numbers on the bottom represent the positions of the CpG dinucleotides relative to the transcriptional start site of the γ-globin gene. Ave indicates the average methylation of the 4 total CpGs. **P < 0.01, Chi-squared test.

In keeping with previous results, the levels of histone mark H4R3me2s on the γ-globin promoter triggered by PRMT5 were significantly reduced in Adox-treated cells compared to untreated cells (Figure 2A). PRMT5 inhibition by Adox treatment followed a dose response (Figure 2B) that occurred over the same drug concentration range as γ-globin induction (Figure 1A).

DNA methylation has been shown to be important in regulation of globin gene expression. Because Adox can inhibit both DNA methylation and protein methylation including histone tail methylation, we performed bisulfite DNA sequencing experiments on globin genes. Using decitabine as a positive control, we found that Adox significantly reduced DNA methylation (Figure 2C). Together these results suggested that Adox was a potent inducer of γ-globin expression in K562 cells.

**Figure 3:**
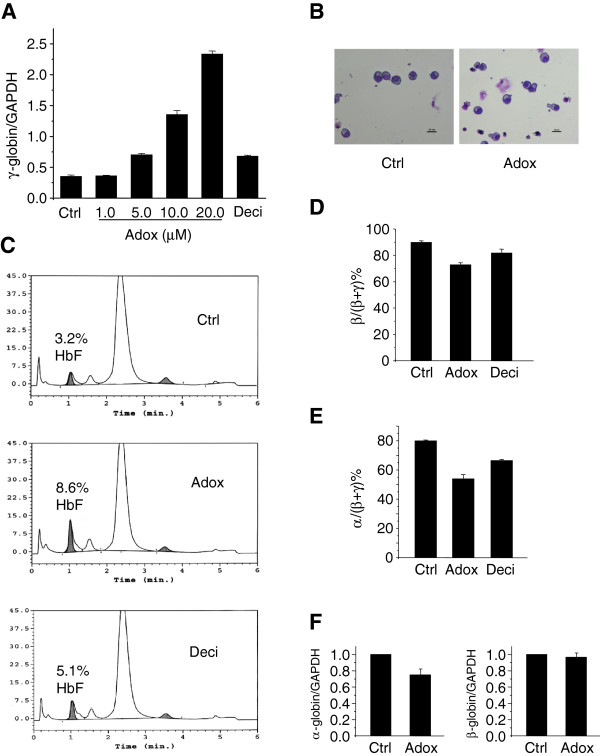
**The effects of Adox to induce fetal globin gene expression in human bone marrow erythroid progenitor cells.** (**A**) Q-RT-PCR quantification of γ-globin from human BM treated with indicated concentrations of Adox or decitabine (Deci, 4 μM) with refreshment twice a week compared to PBS control (Ctrl) after 21 days of culture. Graphs show mean ± SD, n = 3. (**B**) Wright-Giemsa-stained adult bone marrow erythroid progenitor cells at day 21 of differentiation. (**C**) HPLC analysis of globin in human BM cells treated with Adox (20 μM) or decitabine (4 μM). Peaks for HbF are indicated. Data are representative of three independent experiments. (**D**) Q-RT-PCR quantification of mRNA levels of β-globin as percentage of (β-globin + γ-globin) from human BM treated with Adox (20 μM) or decitabine (Deci, 4 μM) with refreshment twice a week compared to PBS control (Ctrl) after 21 days of culture. Graphs show mean ± SD, n = 3. (**E**) Q-RT-PCR quantification of mRNA levels of β-globin as percentage of (β-globin + γ-globin) from human BM treated with Adox (20 μM) or decitabine (Deci, 4 μM) with refreshment twice a week compared to PBS control (Ctrl) after 21 days of culture. Graphs show mean ± SD, n = 3. (**F**) Q-RT-PCR quantification of α-globin and β-globin from BM treated with Adox (20 μM) with refreshment twice a week compared to PBS control (Ctrl) after 21 days of culture. Graphs show mean ± SD, n = 3.

**Figure 4:**
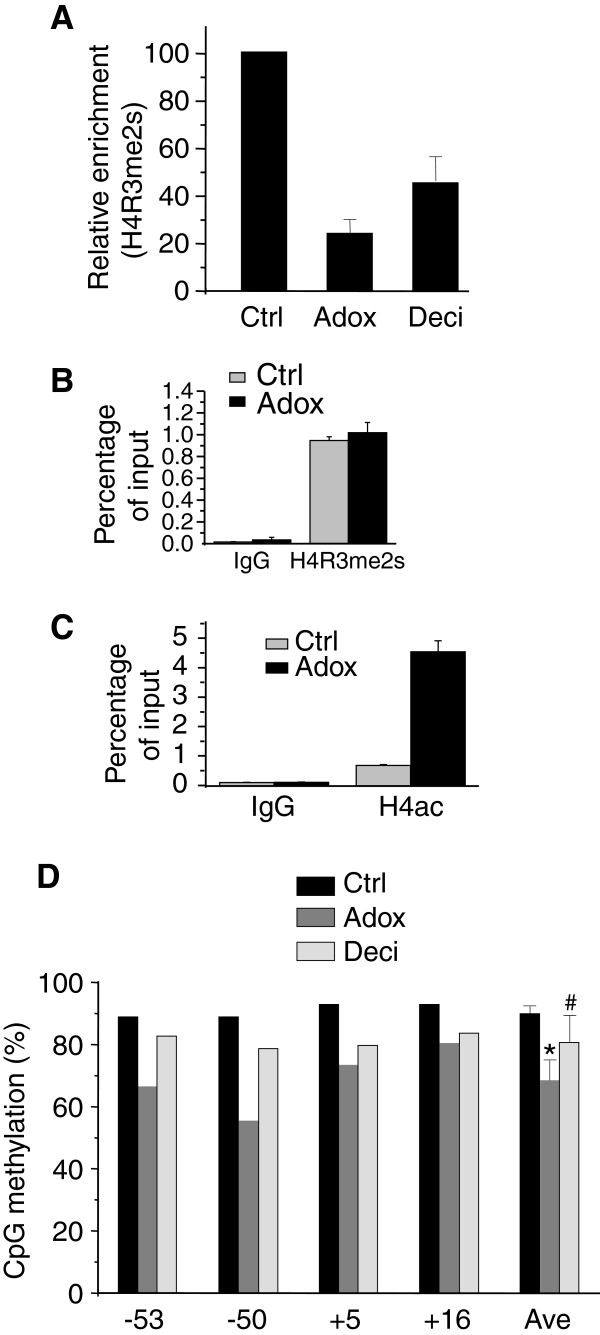
**Modulation of DNA methylation and histone methylation by Adox in human adult erythroid progenitor cells.** (**A**) Histone H4R3me2s ChIP analysis of γ-gene promoter from human BM cells treated with either PBS control, Adox or decitabine as in Figure 3C. Graphs show mean ± SD, n = 3. (**B**) Histone H4R3me2s ChIP analysis of CDH1 promoter from human BM cells treated with either PBS control or Adox as in Figure 3C. Graphs show mean ± SD, n = 3. (**C**) Histone H4 acetylation ChIP analysis of γ-promoter from human BM cells treated with either PBS control or Adox as in Figure 3C. Graphs show mean ± SD, n = 3. (**D**) Quantitation of DNA methylation at the human γ-globin gene in human BM cells treated as in (A). The numbers on the bottom represent the positions of the CpG dinucleotides relative to the transcriptional start site of the γ-gene. Ave indicates average methylation of the 4 total CpGs. *P < 0.05, #P > 0.05 using Chi-squared test.

Next, in order to probe the effect of Adox on human primary erythroid cells, we isolated human bone marrow CD34+ cells and cultured them under optimal conditions for erythroid cell differentiation [11]. We treated human bone marrow cells (BM) with Adox and decitabine (both refreshed twice a week). Total RNA from these cells was isolated and analyzed by Q-RT-PCR. Adox treatment produced a dose–response effect on γ-globin gene expression (Figure 3A). We also confirmed the effect of decitabine on induction of γ-globin gene expression (Figure 3A). We observed that at 20 μM Adox, γ-globin was maximally induced 4-fold relative to the control. No morphological differences of cells were observed during differentiation of Adox treated cells, suggesting that Adox may not perturb overall erythroid differentiation process (Figure 3B). Using HPLC analysis, we confirmed that in human adult bone marrow cells, Adox reactivated HbF to 8.6%, which was 2.7-fold relative to the control, whereas decitabine reactivated HbF to 5.1%, which was 1.6-fold relative to the control (Figure 3C). The α-globin and β-globin of BM cells showed no induction in the presence of either Adox or decitabine (Figure 3D, 3E&3F). Consistent with these results, by ChIP analysis the levels of histone mark H4R3me2s on the γ-globin promoter were greatly reduced in Adox-treated or decitabine-treated BM cells compared to control-treated cells (Figure 4A), but not on CDH1 promoter [14], indicating specificity of Adox for γ-globin gene (Figure 4B). In keeping with this, histone H4 acetylation on the γ-globin promoter was also markedly increased in Adox-treated BM cells compared to the control (Figure 4C). Interestingly, DNA methylation of the γ-globin gene in these BM cells was also reduced, but not as significantly as in Adox-treated K562 cells (Figure 2C &4D). These results suggested that inhibition of histone methylation (H4R3me2s) might be more important than reduction of DNA methylation for inducing fetal globin expression in human bone marrow cells.

Adox is an odorless methyltransferase inhibitor that functions through a feedback loop [15]. Adox can inhibit adenosylhomocysteine hydrolase activity thereby indirectly inhibiting methyltransferases that catalyze adenosylmethionine to adenosylhomocysteine [15]. Mice can tolerate Adox at 100 μmol/kg without any ill-effect [16,17]. Compared to DNA methylation inhibitors such as decitabine, Adox appears to function as an inhibitor of both DNA methylation and protein methylation. It is unclear how this compares to decitabine because we currently do not know the exact mechanism by which decitabine induces γ-globin expression [18].

DNA methylation plays a critical role in modulation of globin gene expression. Inhibitors of DNA methylation or histone deacetylation, such as decitabine and butyrate, have been shown to induce HbF [19,20]. Compared to treatment of K562 cells, treatment of human BM cells with Adox triggered less reduction in methylation at the γ-globin gene, although there was more significant demethylation beyond the transcription initiation site (TIS) at CpG −53 and −50 (Figure 4D). This is similar to results obtained by 5-Azacytidine treatment [18]. The fact that immortalized K562 cells resemble embryonic erythroid progenitors without expression of adult hemoglobin may contribute to this difference. Also, hypermethylation of the γ-globin promoter in BM cells may result in different requirements of Adox concentrations for inducing γ-globin expression in K562 and BM cells. Nevertheless, in this context, DNA hypomethylation produced by Adox treatment may not be a major event or direct trigger in the reactivation of γ-globin expression in human BM cells. Histone modification or repressor complex (e.g. NURD complex) reconstitution which might trigger histone modification changes may contribute to the γ-globin gene reactivation [21-23]. Alternatively, we cannot exclude the possibility that different erythroid specific transcription factors play roles upon γ-globin induction by Adox treatment [24,25].

Histone methylation at H3K9, H3K27, H4K20, or symmetric methylation at H4R3 is normally associated with repression of transcription [26,27]. We have previously demonstrated that histone H4R3me2s is an early histone mark induced by PRMT5 that can coordinately induce other histone methylation events such as H4K20me3, H3K9me3, H3K27me3, and deacetylation of histones [8]. In the current results, in human bone marrow cells, Adox induced γ-globin expression independent of significant hypomethylation of the gene. This suggests that histone methylation, such as H4R3me2s, may play a more critical role in regulation of globin genes.

## Conclusions

Our current studies indicat that Adox reactivates fetal hemoglobin expression efficiently. We speculate that reactivation of fetal globin by Adox may be through a mechanism involving inhibition of PRMT5 in both K562 and human bone marrow erythroid progenitor cells. These findings may contribute to the development of new reagents for reactivating fetal globin expression as a treatment for sickle cell disease and β-thalassemia.

## Competing interests

The authors declare that they have no competing interests.

## Authors’ contributions

YH, GR, and MZ carried out ChIP assays, Northern blot, DNA methylation analyses. JJ, RL, and ZX participated in cell staining, Q-RT-PCR, and Western blot experiments. FB, LC, CM, and RT helped with cell culture and HPLC experiments. SMJ and QZ designed, analyzed the research and wrote the manuscript. All authors read and approved the final manuscript.
